# The Evaluation of the Effects of Dietary Vitamin E or Selenium on Lipid Oxidation in Rabbit Hamburgers: Comparing TBARS and Hexanal SPME-GC Analyses

**DOI:** 10.3390/foods11131911

**Published:** 2022-06-27

**Authors:** Fabiana Trombetti, Paola Minardi, Attilio Luigi Mordenti, Anna Badiani, Vittoria Ventrella, Sabrina Albonetti

**Affiliations:** 1 Department of Veterinary Medical Sciences, University of Bologna, via Tolara di Sopra 50, 40064 Ozzano Emilia, Italy; attilio.mordenti@unibo.it (A.L.M.); anna.badiani@unibo.it (A.B.); vittoria.ventrella@unibo.it (V.V.); sabrina.albonetti@unibo.it (S.A.); 2 Department of Agricultural and Food Sciences, University of Bologna, Viale Fanin 42, 40127 Bologna, Italy; paola.minardi@unibo.it

**Keywords:** rabbit meat, Vitamin E, selenium, lipid oxidation, TBARS, hexanal

## Abstract

The effects and specificity of dietary supplementation of EconomasE^TM^ (EcoE), mainly consisting of organic selenium (0.15 or 0.30 mg/kg feed; Se) or of vitamin E (100 or 200 mg/kg feed; VE), on lipid oxidation were evaluated in rabbit hamburgers during refrigerated storage. Oxidation data obtained by thiobarbituric acid-reactive substances (TBARS) spectrophotometric analysis and solid-phase microextraction (SPME) coupled with gas chromatography (GC) to determine hexanal content were compared. The relationships between oxidation levels, colour and pH and the discriminability of EcoE- or VE-treatment compared with control were also examined. TBARS content decreased in both VE and EcoE groups, while hexanal showed lower values only in the VE100 dietary group. The colour parameters were compatible with acceptable product quality and seemed to parallel the TBARS values up to the second day storage. Both VE and EcoE improved oxidative stability without affecting the sensory properties, but the VE effect appeared to more specifically hamper lipid oxidation, as evidenced by the determination and quantification of hexanal, a specific product of fatty acid peroxidation.

## 1. Introduction

Lipid oxidation is one of the most important processes leading to deterioration in meat quality and is often crucial for food product shelf life [1]. The process is induced by oxygen in the presence of initiators, such as high temperature, light and metal ions and is related to the unsaturation degree of the substrate [2,3]. Therefore, food products with high content of polyunsaturated fatty acids (PUFAs) are more exposed to lipid oxidation. For example, rabbit meat has many desirable nutritional features as a result of its lipid component, characterised by low levels of fat and cholesterol, a high level of unsaturated fatty acids and a good ratio of *n*-6/*n*-3 PUFAs with respect to other types of meat [4,5]. However, the higher amount of PUFAs leads to increasing oxidation with a consequent decrease in product stability during storage and cooking [3,6].

In addition to altering taste and nutritional quality, lipid oxidation involves the formation of reactive and toxic compounds, which may pose a risk to the consumer [2,6]. The major primary products, hydroperoxides, which are essentially odourless, are relatively unstable and decompose into a wide variety of secondary compounds among which aldehydes, in particular malondialdehyde (MDA), are considered to be the most important due to their great contribution to the development of rancid off-flavours and odours [6,7]. One of the major strategies for preventing lipid oxidation in food is the use of antioxidants, and a wide variety of compounds, from synthetic to natural, such as vitamin E (VE) and spices, has been studied [8,9]. However, there is still uncertainty regarding the effectiveness of many antioxidants and the optimal quantity to be used. For example, while the role of VE in stabilising meat colour and reducing lipid oxidative processes has been widely studied and assessed, including in rabbit [4,10,11], the effects of selenium (Se) have been less studied and are still not conclusive. Selenium dietary supplementation has been more extensively studied in cattle, pig and poultry [12,13,14], but only a few studies have considered rabbits, despite their meat susceptibility to lipid oxidation.

Recently, some authors have described a positive effect of Se supplementation on oxidative status and mineral profiles in rabbits [15,16,17], and Minardi et al. [5] found a protective effect against oxidative events in both the *Longissimus dorsi* and *Biceps femoris* muscles. Other authors have also found in pigs a synergistic effect between the administration of Se in the diet and the accumulation of VE in muscles [18]. Additionally in pigs, the synergistic effect between Se and VE in combined dietary administration was also described, where an increase in lipase activity was observed with a consequent enhancement in post mortem lipolysis processes [19]. However, to the best of the authors’ knowledge, the mechanism of action of Se has not yet been fully clarified, and the effects on the oxidative processes have, for the most part, been evaluated by quantifying thiobarbituric acid reactive substances (TBARS).

In terms of the specificity of the oxidative process, the choice of the best oxidation markers is very important for testing the antioxidant capacity and the extent of the lipid substrate oxidation [2]. In the food industry, the most widely used methods measure changes in primary or in secondary products. Therefore, when oxidation occurs at a more accelerated rate, lipid oxidation is best assessed by measuring the secondary products, mainly aldehydes and ketones, due to their quick accumulation [20].

TBARS analysis is one of the oldest and most commonly used methods of assessing lipid oxidation in food, and it is based on the spectrophotometric determination of MDA, a product of oxidation, directly in the food [2]. The TBARS test is often used to evaluate oxidative status owing to its simple procedure and its high correlation with sensory scores [21]; however, it has often been the object of criticism. In fact, MDA only forms from fatty acid chains containing at least three double bonds, such as linolenic acid. In this way, the decomposition products of the peroxidation of linoleic and oleic acid are excluded. Moreover, the TBARS test is not specific for MDA since thiobarbituric acid (TBA) can react with other aldehydes, browning reaction products, protein and sugar degradation products, amino acids and nucleic acids [2]. The TBARS test can be used to assess the extent of oxidation rather than to quantify MDA [1,22].

In recent years, different methods of recovering secondary products have been proposed. Some of these products are considered to be highly specific markers of the lipid oxidation of a particular polyunsaturated fatty acid family. For instance, propanal is regarded as the main oxidation marker of *n*-3 fatty acids, while hexanal and pentanal are reputed to be good oxidation markers of *n*-6 fatty acids. In particular, hexanal, as its formation is usually greater than that of other secondary products, is the most used marker for lipid oxidation [6]. In several studies on food, solid-phase microextraction coupled with gas chromatography (SPME/GC) has been used to detect lipid oxidation products such as hexanal [1,22,23,24]. Although the use of this technique is increasing due to its sensitivity and simplicity, it is not always reported as the most suitable technique for detecting the extent of lipid oxidation, and it is still controversial as a replacement for the TBARS test. Some studies have found a perfect correlation between the SPME/GC technique and that of TBARS [22], while in other cases, TBARS proved to be more sensitive and accurate oxidation markers [1].

In this work, the effects of two antioxidants, VE and EconomasE^TM^ (EcoE), on lipid oxidation are considered by comparing two different analytical methods. Hence, the effect of EcoE, a patented premixture of nutritional additives consisting mainly of Se, was compared with that of VE in rabbit hamburgers during refrigerated storage. The study of antioxidant supplementation appears to be of great interest, in particular in meat preparations because they might be more easily exposed to oxidative processes as well as bacteria contamination, and cold storage is not always sufficient to contain these problems. The choice of rabbit meat was related to its valuable nutritional characteristics that, due to its greater richness in PUFAs, make it more susceptible to oxidative processes; moreover, rabbit meat has been studied much less than other meats despite its nutritional value and its increased use in recent years [25].

In particular, the principal aim of this study was to evaluate the effects and the specificity of VE or Se dietary supplementation on the lipid stability of rabbit hamburger meat during refrigerated storage, measuring the secondary products of oxidation through TBARS spectrophotometric analysis and the SPME/GC determination of the hexanal content.

In parallel, the possible relationship between the data related to the peroxidation products and those related to meat colour and pH, quality parameters often related to oxidation processes, was considered. The sensory discriminability among cooked rabbit hamburgers obtained from the different dietary groups was also assessed using triangle tests.

## 2. Materials and Methods

### 2.1. Animals and Diets

The Scientific Ethics Committee on Animal Experimentation approved the experimental protocol (University of Bologna, Prot. ID 1/72/2012).

For the analyses, 270 commercial New Zealand white male rabbits (*Oryctolagus cuniculis*), provided by the Rabbit Genetic Centre of the Martini Group (Budrio di Longiano, FC, Italy), were selected and randomly divided into 5 dietary groups of 54 animals each; these were subdivided into 3 groups with ad libitum access to water and feed, as reported by Albonetti et al. [26] and Minardi et al. [5].

Both the starter basal diet (SBD) and the finisher basal diet (FBD) used in the first (5–8 weeks) and second (9–12 weeks) periods, respectively, were formulated to meet the nutrient requirements of the animals during the experimental period and were supplemented with 50 mg/kg of VE (Sigma-Aldrich, St. Louis, MO, USA) and 0.22 mg/kg of sodium selenite (Na_2_SeO_3_) corresponding to a Se amount of 0.099 mg/kg of feed. Ingredients and compositions of the two basal diets are shown in Table 1 and detailed in Minardi et al. [5].

The basal diets of two groups were supplemented with 100 or 200 mg/kg of dl-α-tocopheryl acetate (Sigma-Aldrich, St. Louis, MO, USA) (VE 100 and VE 200 groups, respectively). The diets of another two groups were supplemented with 100 or 200 mg/kg of EcoE (Alltech Nicholasville, KY, USA) (EcoE 100 and EcoE 200 groups, respectively), as suggested by the producer. The remaining group was fed a normal diet and used as a control (CTRL). The organic Se in the EcoE premixture corresponded to a Se amount of 1500 mg/kg of feed; therefore, the 100 and 200 mg/kg added to the EcoE100 and EcoE200 diets contained 0.15 mg and 0.30 mg/kg of Se, respectively. In the EcoE premixture, the amount (50,000 mg/kg) of vitamin C (VC) corresponded to 5 and 10 mg/kg of feed in the EcoE100 and EcoE200 diets, respectively. The small amount of VC present in EcoE, lower than the minimum requirements for rabbits [21], suggests excluding that any effects found with the administration of EcoE could be attributed to VC.

The contents of α-tocopheryl acetate and Se in feed are reported in Table 2 as previously determined by Minardi et al. [5].

At 12 weeks of age, the rabbits were transported to the slaughterhouse (Ma.Ge.Ma abattoir, Savignano sul Rubicone, FC, Italy), weighed and electrically stunned (70 V DC, 50 Hz, 5 s). After complete bleeding, skin and viscera were removed, and the hot carcasses were weighed and then cooled at 4 °C for 24 h in a refrigerated cell (Costan Daily TN SP60/4, Costan S.p.A., Belluno, Italy). Ten carcasses were randomly selected from each dietary group for analysis. Carcass hygiene was tested, as reported in detail in Albonetti et al. [26], and the meat was used to prepare hamburgers.

### 2.2. Rabbit Hamburgers

Carcasses from each dietary group were boned, and the meat was minced twice using a refrigerated mincer (TC 32 Frozen, Sirman Spa, Padova, Italy) to generate a single batch of minced meat from which all the necessary hamburgers were produced, without any seasoning or additives in order to avoid interference with the tested antioxidants.

The hamburgers (100 ± 3 g; 3 for each group and for each sampling time) were immediately prepared, using a conventional burger maker having an average dimension of 10 cm in diameter and 1 cm in thickness, and were wrapped with plastic food film. A part of the minced meat, after being placed in a plastic bag and subjected to vacuum treatment, was stored at −20 °C and was used for sensory analysis as described in the specific section.

The hamburgers were stored at 0–2 °C in a refrigerator (Quartet 200, Costan, Belluno, Italy) which was closed with a lid every night to reproduce retail storage conditions. Immediately after production (time 0, t0) and after 1, 2 and 4 days (t1, t2 and t4, respectively), the hamburgers were analysed for pH and colour or were stored in liquid nitrogen if designated for lipid oxidation analyses. Preliminary tests had shown that this storage method effectively protects against oxidative processes.

The contents of α-tocopherol and Se in minced meat, previously determined by Minardi et al. [5], are reported in Table 2. The Se content in meat burgers of EcoE200 groups was 20.3 µg/100 g, well below the minimum daily requirement for humans (55 µg/100 g) [27].

### 2.3. Oxidation Products Analysis

The occurrence of secondary oxidation products in hamburgers was checked using two different approaches, which complemented each other, i.e., the analysis of TBARS and the determination of the hexanal.

#### 2.3.1. TBARS Analysis

The TBARS were determined using a colorimetric assay [28] as equivalents of MDA, one of the low-molecular-weight products formed by lipid peroxidation [29], using a UV-Vis Spectrophotometer Perkin Elmer Lambda 45.

The samples (2 g) were mixed with 20 mL of ultrapure water and homogenized using an Ultraturrax (IKA) for 30 s at 13,500 rpm. Aqueous trichloroacetic acid (25% *w/v*) was added to a 5% final concentration (*v/v*), and the samples, after stirring at 4 °C for 15 min, were centrifuged for 15 min at 11,500 rpm. An aliquot (2.1 mL) was pipetted from the supernatant into a screw-capped tube, and 0.9 mL of 0.6% *w/w* 2-TBA water solution was added. The mixture was incubated at 70 °C for 30 min and then cooled under tap water. The TBARS levels were estimated at 535 nm using malonaldehyde bis(dimethyl acetal) (1,1,3,3,tetrametoxypropan) as standard. The concentration of MDA in the samples was calculated on the basis of the slope and intercept data of the computed least-squares fit of the calibration curve.

#### 2.3.2. Hexanal Analyses

The analyses to determine hexanal content were carried out on homogenised samples (1.0 g) put into 10 mL glass cylindrical auto-SPME vials (Supelco^®^) to which a NaCl saturated solution was added to reach 5 mL [30]. The vials, immediately sealed with an assembled aluminium screw cap with a hole and a polytetrafluoroethylene/silicone septum, were stirred for 1 min at room temperature, conditioned at 40 °C for 30 min and then placed in an autosampler at 50 °C for 5 min for absorption of the headspace volatiles using SPME. A mixed SPME fibre 50/30 µm divinylbenzene-carboxen-polidimethylsiloxane (DVB/CAR/PDMS, Supelco^®^) was used to extract the headspace volatiles. The target analyte on the loaded fibre was thermally desorbed for 5 min at 250 °C directly in the GC injector port, used in splitless mode. Both the absorption and the desorption phases were controlled by the Varian CP-8200 autosampler. The GC analyses were carried out on a Varian 3380 GLC equipped with a fused silica capillary column Equity-5 Supelco (30 m × 0.25 mm i.d. and film thickness 0.25 µm) and a flame ionisation detector (FID). The chromatographic conditions were: H_2_ as a carrier gas (20 mL/min); temperature program: 35 °C was held for 5 min, 8 °C min^−1^ to 75 °C, 40 °C min^−1^ to 200 °C and final isotherm at 200 °C for 5 min; the FID was held at 250 °C.

The data were processed using a Varian Star Chromatography Workstation. The identification of hexanal levels was carried out using internal standards. An external calibration procedure was used to quantify the extracted amount of target analyte from the SPME fibres, with a calibration range from 50 to 1000 ng. Linear concentration–response relationships were always obtained with high confidence (*r*^2^ > 0.95).

### 2.4. pH and Colour Evaluation

The ultimate pH (pHu) was measured using a pH-meter (HI92240, Hanna Instruments, Padova, Italy) with a penetration electrode (Double-Pore cod. n° 32384003, Hamilton). The pH probe was calibrated using two buffers (pH 4.01 and 7.01, HI5400-12 and HI5700-12, respectively, Hanna Instruments, Villafranca Padovana, Italy), and each measurement was performed three times.

The colour parameters (L*, a*, b*) were evaluated using a tristimulus analyser equipped with an 8 mm diameter measuring area (Minolta Chroma-Meter CR-200, Minolta Inc., Osaka, Japan) according to the CIE LAB approach [31], selecting D65 as illuminant and a 0° viewing angle, as mentioned by Honikel [32].

### 2.5. Sensory Evaluation

Triangle tests [33] were carried out to determine whether plain consumers could detect any difference between the CTRL and VE 200 or CTRL and EcoE 200 dietary groups. The highest concentration of antioxidant was selected because it was thought that any effect on the sensory response would be more discernible. Moreover, in this way, at each detection time, it was possible to halve the number of consumers required to implement a “proper” triangle test, i.e., using a brand-new set of consumers with no previous experience with rabbit meat supplemented with antioxidants. In detail, on six distinct occasions (at 15, 30, 60, 120 and 240 days from the date of pouch preparation), six different groups of 18–20 consumers, as detailed in Section 3.3, all regular consumers of rabbit meat and all volunteers, convened in three separate shifts in a rather large room. That room was both olfactorily and visually neutral and temporarily arranged with 6–7 individual booths kept in dim light. Each panel was composed of students and staff at the Department of Veterinary Medical Sciences - DIMEVET (overall gender ratio, male proportion = 0.474; age ranging from 24 to 67 years). Each consumer received a triangle test [32] arranged on a separate tray, prepared with meat patties presenting either the CTRL vs. VE200, or the CTRL vs. EcoE200 dietary groups to be compared, as detailed below.

Ground meat from each dietary group had been prepared as 30 g patties, each of which was put into an uncovered glass baby food jar and microwaved at 750 W (cooking time = 120 + 120 + 60 s) in a 2450 MHz, 1000 W variable power oven Mod. MT 243/486, (Whirlpool Europe, S.r.l., Comerio, Italy). Each jar had been covered with its own lid to keep its contents as warm as possible and then randomly assigned a three-digit number. Each triangle test consisted of three jars (the contents of two of which were identical while the third was different) placed in a line on the same tray. The panellists were asked to identify the odd sample, having been previously instructed to cleanse their palate between samples with the soft part of sliced unsalted Tuscan bread and still water at room temperature. In addition, as suggested by the relevant International Organization for Standardization (ISO) norm [33], the consumers were asked to briefly comment as to why their choice of the odd sample had been made.

### 2.6. Statistics

The data were reported as mean values ± standard deviation (SD) of at least three determinations. To compare the differences between the dietary experimental groups and between storage times, two-way analysis of variance (ANOVA) was carried out on all data. The Tukey test was used to determine if the differences in the mean values were statistically significant (*p* ≤ 0.05). Before the statistical analyses, the data had been checked for normal distribution and variance homogeneity. All the statistical analyses were carried out using SigmaStat^®^ release 2.0 (SPSS Inc., Chicago, IL, USA).

As for sensory evaluation [33], a significance level of *p* ≤ 0.05 was selected to determine whether the number of correct answers was sufficient as given, each time, by a panel of 18–20 plain consumers.

## 3. Results and Discussion

The hamburgers were tested up to the fourth day of storage because, after that, they had clearly deteriorated as to odour and appearance, and as such, they were no longer suitable for commercialisation.

### 3.1. Lipid Oxidation

In meat and meat products, TBARS and hexanal content are generally considered to be reliable indices for evaluating the extent of lipid oxidation and, thus, the development of oxidative “off flavour” (i.e., rancidity), which is recognised as a serious problem during the storage of meat products. However, TBARS testing has been the object of criticism because it is not specific enough to quantify lipid peroxidation products, as previously reported [1,22].

In the present study, the results showed that dietary supplementation with VE or EcoE significantly affected the TBARS production (Table 3). Indeed, the two different dietary treatments, VE and EcoE, at both the tested concentrations significantly decreased TBARS content with respect to the basal diet, although with some differences. This beneficial effect disappeared over a storage time of 4 days (t4) in the EcoE 200 group and in the VE groups. In the Eco100 group, the beneficial effect disappeared at t2.

The lower TBARS content in all the treated groups with respect to the control group, already perceivable at t0, could be considered the first positive outcome of dietary supplementation with VE or Se instead of their simple addition to meat as preservatives. The decrease in TBARS is particularly evident in the group treated with VE 200, where the values are more than halved compared with the control (about 45% compared with the control). Compared with raw meat, processed meat such as hamburgers is more susceptible to oxidative damage due to physical manipulation during the manufacturing process, involving the disruption of muscle structure and the exposure of muscle lipids to an oxidative environment. In this case in particular, dietary VE deposited into subcellular structures becomes an integral part of muscle and fat tissue, thus becoming very effective against lipid oxidation as previously reported [34]. In fact, Minardi et al. [5] recently reported that dietary supplementation with VE, at the same concentrations used in the present work, increased the content of this antioxidant in the same meat preparation (Table 2, “Materials and Methods” section).

Hexanal, being a specific indicator of the oxidation of important long-chain unsaturated fatty acids, in particular ω-6 PUFA, has been reported to be a valid and specific indicator of oxidative stability [35], and, thus, it could be used as a marker of lipid oxidative processes regarding hamburger shelf-life. In this study, however, the richness of ω-6 fatty acids both in diets and in meats, highlighted in the previous study by Minardi et al. (2020), makes hexanal a good marker for studies relating to lipid oxidation.

Hexanal values, reported in Table 4, showed trends only partially similar to those of TBARS.

The detected hexanal values were similar in all the experimental groups and, except for the VE100 dietary group, only slightly affected by dietary treatment. In the VE100 dietary group, on the other hand, the hexanal content was significantly lower, approximately 35%, than that in the CTRL group from t0 to t4. The absence of effect of VE at concentrations above 100, corresponding to the amount of 4.22 mg/kg in meat, reveals that an optimal intake of VE is necessary for the best antioxidant effect, as also observed by other authors, who found better protective effects at concentrations below 200 [10,36,37,38]. The significant decrease in hexanal content at t4 in the CTRL and VE200 dietary groups could suggest that lipid oxidation had entered a termination stage as recently suggested [39]. The data regarding the VE dietary groups seemed to confirm the protective effect of VE on lipid oxidative stability found in other studies [4,5,40,41] in which the reduced oxidation was sometimes also accompanied by an increase in VE content in the muscle [5,19,40]. The effects described for VE usually referred to a specific action on lipid oxidation; in fact, due to its lipophilic structure, VE is considered to be a potent, and probably the most important, lipophilic antioxidant [42]. On the other hand, the hexanal values found for EcoE groups, which were not different from those for the control group, do not seem to confirm a specific protective effect of the dietary supplementation with Se on lipid oxidation. Minardi et al. [5] have recently found a protective effect of organic Se on oxidative stability, with enriched Se content in *Longissimus lomborum* and *Biceps femoris* rabbit muscles (Table 2). A similar protective effect was also observed in some other studies involving rabbits [15,17] and other species [12,13,43]. In particular, Papadomichelakis et al. [15] demonstrated that dietary organic Se supplementation, from 0 to 2.5 mg/kg, increases the Se content of rabbit meat and supplementation with 0.5 mg/kg Se improved oxidative stability, while at the highest doses, a prooxidant effect was observed. Similarly, Mattioli et al. [17] found that diet supplemented with 10% olive leaves enriched in Se (2.17 mg of Se per kg of dried leaves) positively affected the oxidative status of rabbit meat, reducing the TBARS value and carbonyl derivatives of proteins. An antioxidant effect was found in rabbits also with the supplementation of Nano–Se or sodium selenite [16]. On the other hand, some authors have found that Se supplementation did not affect oxidative meat stability [14,44,45] or have described a positive Se effect in retarding oxidation processes in plasma or liver but not in meat [41,46]. However, to the best of the authors’ knowledge, the effects of Se on oxidative processes have been evaluated mainly through TBARS analysis.

In the present study, the comparison of the results obtained from the two parameters of lipid peroxidation, TBARS and hexanal, seemed to highlight the following main aspects. In general, both methods could give a good indication of the oxidation processes, which seemed to be reduced by the presence of the antioxidants administered in the diet. In particular, the effects of the antioxidants were highlighted more clearly by the TBARS data, while the hexanal data showed a containment effect of the degradative processes only in the VE 100 dietary group. This could have indicated a more specific correlation of this index with the lipid oxidative processes. In fact, hexanal is a typical and well-known end product of the oxidation of fatty acids, in particular *n*-6. The values found for TBARS, which other authors have also considered to be less specific in detecting the presence of oxidative processes [2], could in fact be due to the simultaneous evolution of other degradative processes, including those related to proteins, as also described by other authors [6]. As is known, for example, the Maillard reaction leads to the production of carbonyl compounds. Moreover, the advances in meat science research have indicated that the same oxidants which initiate lipid oxidation can cause and propagate protein oxidation, and carbonyl formation is a common reaction pathway of the oxidation process. Moreover, recent studies have shown that MDA, as another by-product of lipid oxidation, can affect heme pigment, protein carbonylation and reactive oxygen species-generating system, promoting myofibrillar oxidation [47,48]. In addition, protein oxidation occurs by means of a chain reaction of free radicals, such as the oxidation of lipids in animal muscle [11,49].

Hence, the almost complete absence of variation in the hexanal content in the EcoE dietary groups could have indicated that Se dietary supplementation could prevent the degradative processes but with a more extensive effect and not specifically directed only to lipid oxidation. Moreover, Se intake could modulate the antioxidant enzyme glutathione peroxidase, thus contributing to the overall antioxidant defence of muscle, as has also been suggested in other studies [11].

### 3.2. Meat Quality

Some parameters relating to meat quality studied and data are reported in Table 5. It is widely accepted that lipid and protein oxidation, in particular myoglobin oxidation, greatly affect meat quality, but the impacts of oxidation reactions on meat colour and discoloration are still controversial. As for the pH values, they were similar to those reported by Paci et al. [50] in rabbit meat and higher than those determined in raw rabbit hamburgers by Mancini et al. [51]. The differences occasionally detected between the dietary groups and at the various storage times were not relevant and were hardly ascribable to a diet effect. However, the pH values observed for the VE100 dietary group appeared to be the lowest during the time period considered.

Instrumental measurements of colour only occasionally showed significant differences, not always easily correlated to the different dietary treatments or to the storage time, especially for the L* values, which in fact remained essentially the same over time in all the dietary groups with the exception of the control group; in that group, starting from t2, a slight lowering was observed.

A decreasing trend in a* values at t4 was observed in all the dietary groups, independent of diet supplementation. As for the b* values, a similar decreasing trend was observed. The variations in the colour parameter, both influenced to redox state of heme pigment, could have been related to the oxidation of the hamburger, as also reported by Mancini et al. [51], since it paralleled the strong increase in MDA content. In particular, the variations in the a* values observed in this study were similarly reported by Georgantelis et al. [52] in beef hamburgers and were attributed to the gradual oxidation of myoglobin and the simultaneous accumulation of metmyoglobin over time [53,54]. Similar trends and observations were reported by Wang et al. [55], who clearly demonstrated that myoglobin and lipid oxidation inversely impacted redness. At least up to t2, the data of the colour parameters appeared compatible with an acceptable product quality level and seemed to parallel the TBARS values, which were above 1 and close to 1.5 only at t4, denoting the rancidity of the product [56].

### 3.3. Sensory Evaluation

The results obtained from the triangle tests gave evidence that no detectable difference between either the CTRL and the VE 200 dietary groups, or the CTRL and the EcoE 200 dietary groups emerged over eight months of frozen storage (Table 6).

## 4. Conclusions

This study confirmed the antioxidative effects of VE or EcoE when added to rabbit diet, in concentrations compatible with humans’ nutritional needs, without affecting the sensory characteristics of the meat or meat products. In fact, dietary supplementation with antioxidants improved the stability of the product by reducing degradation processes with respect to the control group. While the VE protective effect appeared more directed to lipid oxidation, already at the lowest concentration—probably due to its lipophilic features—the effect of Se seemed less specific and probably directed at preventing both lipid and protein deterioration. Therefore, the quantification of hexanal, one of the main and specific markers of lipid peroxidation, the increase of which is contained only by the VE at the lower concentration, could be useful and reliable for studying oxidative processes involving lipid components and shoulder the more specific antioxidant effect of VE on lipid oxidation.

## Figures and Tables

**Table 1:** Ingredients and chemical composition of the starter and finisher rabbit basal diets (Data from Minardi et al. [5]).

Item	Basal Diets ^1^	
	SBD	FBD
Ingredients (g/kg diet)		
Wheat bran	250	160
Sunflower seed	195	215
Sugar beet pulp	150	150
Alfalfa hay	100	90
Barley	NP	85
Alfalfa dehydrated	80	30
Wheat flour middlings	50	70
Sugarcane molasses	50	50
Grape seeds meal	NP	50
Oats	40	40
Olive-pomace oil	35	NP
Calcium carbonate	12	8
Soybean mill run	NP	20
Palm oil	8	11
Sodium chloride	4	4
Soybean oil	3.3	3.3
Supplement	22.7	13.7
Vitamin E	0.05	0.05
Selenium (mg/kg)	0.1	0.1
Chemical composition (g/kg as feed) ^2^		
Dry matter	885.1 ± 1.2	890.5 ± 0.6
Crude protein	164.3 ± 0.9	164.1 ± 0.9
Ether extract	42.6 ± 0.5	47.5 ± 0.4
Crude fiber	194.5 ± 4.4	160.5 ± 1.9
Starch	153.0 ± 5.3	228.4 ± 6.7
Ash	105.9 ± 1.0	81.5 ± 0.4
Neutral detergent fiber	411.4 ± 5.2	329.8 ± 2.9
Acid detergent fiber	271.7 ± 5.1	209.6 ± 1.7
Acid detergent lignin	71.7 ± 2.1	69.3 ± 3.1
Ca	17.8 ± 0.5	13.4 ± 0.2
P	5.5 ± 0.1	6.5 ± 0.1
Gross energy (GE) MJ/kg	16.11 ± 0.2	16.65 ± 0.2

NP = not present. ^1^ SBD = starter basal diet used in the first period (5–8 weeks); FBD = finisher basal diet used in the second period (9–12 weeks). ^2^ *n* = 3; data are expressed as means ± standard error.

**Table 2:** The contents of α-tocopheryl acetate and selenium in feed and α-tocopherol and selenium in minced meat (Data from Minardi et al. [5]).

Item ^1^		Dietary Groups ^2^					SEM ^3^	*p* Value
		CTRL	VE100	VE200	EcoE100	EcoE200		
αT acetate (mg/kg)	Feed	85.00 ^a^	132.40 ^b^	253.00 ^c^	90.00 ^a^	86.80 ^a^	5.21	<0.001
αT (mg/kg)	Meat	2.78 ^a^	4.22 ^b^	4.60 ^b^	3.04 ^a^	3.16 ^a^	0.27	<0.001
Se (mg/kg)	Feed	0.096 ^a^	0.094^a^	0.095 ^a^	0.250 ^b^	0.395 ^c^	0.042	<0.001
Se (mg/kg)	Meat	0.174 ^a^	0.165 ^a^	0.175 ^a^	0.191 ^b^	0.203 ^b^	0.015	<0.001

^1^ αT = α-tocopherol; αT acetate = α-tocopheryl acetate; Se = selenium. ^2^ CTRL = control diet (BD: basal diet); VE100 = BD supplemented with 100 mg of vitamin E per kg of feed; VE200 = BD supplemented with 200 mg of vitamin E per kg of feed; EcoE100 = BD supplemented with 100 mg of EconomasE^TM^ per kg of feed; EcoE200 = BD supplemented with 200 mg of EconomasE^TM^ per kg of feed. 3 SEM = standard error of the mean. Values are given as means (*n* = 5). Different letters within the same row indicate significant differences (*p* < 0.05) ^(a,b,c)^ using a one-way ANOVA, Student–Newman–Keuls test.

**Table 3:** The effects of dietary supplementation with α-tocopherol acetate (VE) or EconomasE^TM^ (EcoE) and storage time on the TBARS (mg MDA/kg product) of packaged rabbit hamburgers.

Dietary Groups		TBARS (mg MDA/kg Product)				
		CTRL	VE100	VE200	EcoE100	EcoE200
Storage time	t0	0.751 ^ax^ ± 0.026	0.408 ^bx^ ± 0.007	0.347 ^bx^ ± 0.029	0.501 ^bx^ ± 0.070	0.344 ^bx^ ± 0.039
	t1	0.773 ^ax^ ± 0.010	0.477 ^bx^ ± 0.155	0.376 ^bx^ ± 0.092	0.479 ^bx^ ± 0.024	0.522 ^bx^ ± 0.114
	t2	0.775^ax^ ± 0.016	0.431 ^bx^ ± 0.036	0.335 ^bx^ ± 0.046	1.091 ^cy^ ± 0.130	0.515 ^bx^ ± 0.039
	t4	1.445 ^y^ ± 0.023	1.312 ^y^ ± 0.275	1.359 ^y^ ± 0.062	1.273 ^y^ ± 0.174	1.373 ^y^ ± 0.127

Values are given as mean ± standard deviation (SD) of at least three determinations. ^a,b,c^ Different letters within the same row indicate significant differences (*p* ≤ 0.05) between groups on the same storage day. ^x,y^ Different letters within the same column indicate significant differences (*p* ≤ 0.05) between storage days in the same dietary group.

**Table 4:** The effects of dietary supplementation with α-tocopherol acetate (VE) or EconomasE^TM^ (EcoE) and storage time on the hexanal content (mg/kg product) of packaged rabbit hamburgers.

Dietary Groups		Hexanal (mg Hexanal/kg Product)				
		CTRL	VE100	VE200	EcoE100	EcoE200
Storage time	t0	0.477 ^ax^ ± 0.026	0.154 ^by^ ± 0.009	0.571 ^ax^ ± 0.009	0.514 ^ax^ ± 0.014	0.573 ^ax^ ± 0.094
	t1	0.535 ^ax^ ± 0.103	0.255 ^by^ ± 0.048	0.450 ^ax^ ± 0.056	0.404 ^cy^ ± 0.148	0.585 ^ax^ ± 0.013
	t2	0.555 ^ax^ ± 0.080	0.165 ^by^ ± 0.044	0.531 ^ax^ ± 0.081	0.536 ^ax^ ± 0.044	0.618 ^ax^ ± 0.033
	t4	0.193 ^by^ ± 0.006	0.238 ^by^ ± 0.037	0.287 ^by^ ± 0.062	0.913 ^dz^ ± 0.035	0.605 ^ax^ ± 0.009

Values are given as mean ± standard deviation (SD) of at least three determinations. ^a,b,c,d^ Different letters within the same row indicate significant differences (*p* ≤ 0.05) between groups on the same storage day. ^x,y,z^ Different letters within the same column indicate significant differences (*p* ≤ 0.05) between storage days in the same dietary group.

**Table 5:** The effects of dietary supplementation with α-tocopheryl acetate (VE) or EconomasE^TM^ (EcoE) and storage time on the pH and colour parameters (L*, a*, b*) of packaged rabbit hamburgers.

Parameter	Storage Time	Dietary Groups				
		CTRL	VE100	VE200	EcoE100	EcoE200
pH	t0	5.93 ^by^ ± 0.05	5.90 ^bx^ ± 0.16	5.88 ^bx^ ± 0.05	6.00 ^bx^ ± 0.07	5.94 ^by^ ± 0.03
	t1	5.88 ^aby^ ± 0.02	5.79 ^ax^ ± 0.03	5.89 ^abx^ ± 0.02	5.98 ^bx^ ± 0.02	5.92 ^by^ ± 0.06
	t2	5.96 ^by^ ± 0.05	5.74 ^ax^ ± 0.03	5.71 ^ay^ ± 0.03	5.70 ^ay^ ± 0.04	5.86 ^by^ ± 0.03
	t4	6.00 ^by^ ± 0.04	5.83 ^ax^ ± 0.06	5.85 ^ax^ ± 0.02	5.91 ^by^ ± 0.10	5.92 ^by^ ± 0.06
L*	t0	56.31 ^ax^ ± 2.33	54.87 ^ax^ ± 2.70	53.23 ^ax^ ± 0.52	51.25 ^b^ ± 0.65	55.02 ^a^ ± 1.76
	t1	56.17 ^ax^ ± 0.92	54.41 ^ax^ ± 1.03	53.05 ^ax^ ± 0.37	52.12 ^b^ ± 0.91	54.10 ^a^ ± 0.33
	t2	50.72 ^y^ ± 0.50	51.86 ^x^ ± 1.10	51.16 ^x^ ± 1.18	51.78 ± 1.06	54.06 ± 0.40
	t4	50.73^by^ ± 3.02	52.10 ^ax^ ± 3.02	54.28 ^ax^ ± 2.98	54.65 ^a^ ± 1.55	55.12 ^a^ ± 1.71
a*	t0	7.53 ^x^ ± 1.95	9.20 ^x^ ± 0.24	6.64 ^xy^ ± 0.62	8.23 ^x^ ± 1.21	7.51 ^x^ ± 1.50
	t1	6.94 ^x^ ± 1.62	7.02 ^xy^ ± 1.22	8.25 ^x^ ± 0.70	8.37 ^x^ ± 1.56	7.26 ^x^ ± 0.75
	t2	7.21 ^x^ ± 0.58	7.75 ^xy^ ± 0.87	6.98 ^x^ ± 0.57	7.85 ^x^ ± 0.79	5.84 ^y^ ± 0.73
	t4	4.59 ^y^ ± 0.61	5.52 ^y^ ± 0.58	4.37 ^y^ ± 0.62	4.77 ^y^ ± 1.58	4.68 ^y^ ± 1.69
b*	t0	7.11 ^x^ ± 1.18	7.97 ^x^ ± 0.93	6.69 ^xz^ ± 0.28	7.81 ^x^ ± 1.00	7.57 ^x^ ± 0.44
	t1	8.66 ^x^ ± 1.93	7.14 ^x^ ± 0.66	8.35 ^x^ ± 0.52	8.26 ^x^ ± 0.52	8.68 ^x^ ± 1.16
	t2	5.52 ^y^ ± 0.84	4.87 ^y^ ± 0.62	4.42 ^y^ ± 0.04	5.75 ^y^ ± 0.45	5.46 ^y^ ± 0.21
	t4	4.30 ^y^ ± 0.73	4.46 ^y^ ± 0.38	5.76 ^yz^ ± 0.71	5.63 ^y^ ± 0.46	5.45 ^y^ ± 0.79

Values are given as mean ± standard deviation (SD) of at least three determinations. ^a,b^ Different letters within the same row indicate significant differences (*p* ≤ 0.05) between groups on the same storage day. ^x,y,z^ Different letters within the same column indicate significant differences (*p* ≤ 0.05) between storage days in the same dietary group.

**Table 6:** Diet supplementation discriminability via triangle test. Number of correct identifications (CI) of diet are reported.

	Storage Days	15		30		60		120		240	
		(18 Consumers)		(20 Consumers)		(18 Consumers)		(18 Consumers)		(18 Consumers)	
		CI	*p*	CI	*p*	CI	*p*	CI	*p*	CI	*p*
**Diet**											
**CTRL**		3	n.s.	5	n.s.	1	n.s.	4	n.s.	1	n.s.
**VE200**		1	n.s.	2	n.s.	3	n.s.	0	n.s.	0	n.s.
**EcoE200**		1	n.s.	2	n.s.	4	n.s.	2	n.s.	4	n.s.

n.s. = not significant. The dietary inclusion of up to 200 mg/kg of either VE or EcoE in the diet did not seem to generate any perceivable sensory difference in the related meat.

## Data Availability

The data presented in this study are available on request from the corresponding author.
